# A synthesis of the arctic terrestrial and marine carbon cycles under pressure from a dwindling cryosphere

**DOI:** 10.1007/s13280-016-0872-8

**Published:** 2017-01-23

**Authors:** Frans-Jan W. Parmentier, Torben R. Christensen, Søren Rysgaard, Jørgen Bendtsen, Ronnie N. Glud, Brent Else, Jacobus van Huissteden, Torsten Sachs, Jorien E. Vonk, Mikael K. Sejr

**Affiliations:** 1 Norwegian Institute of Bioeconomy Research, Høyskoleveien 7, 1430 Ås, Norway; 2 Department of Physical Geography and Ecosystem Science, Lund University, Sölvegatan 12, 223 62 Lund, Sweden; 3 Centre for Earth Observation Science (CEOS), Clayton H. Riddell Faculty of Environment Earth and Resources, University of Manitoba, 440 Wallace Building, Fort Gary Campus, Winnipeg, MB R3T 2N2 Canada; 4 Arctic Research Centre, Aarhus University, Ny Munkegade 114, bldg. 1540, 8000 Aarhus C, Denmark; 5 Greenland Institute of Natural Resources, Kivioq 2, Box 570, 3900 Nuuk, Greenland; 6 ClimateLab, Symbion Science Park, Fruebjergvej 3, Boks 98, 2100 Copenhagen O, Denmark; 7 Department of Biology, Nordic Center for Earth Evolution, University of Southern Denmark, Campusvej 55, 5230 Odense M, Denmark; 8 Department of Geography, University of Calgary, 2500 University Dr. NW, Calgary, AB T2N 1N4 Canada; 9 Vrije Universiteit, Faculty of Earth and Life Sciences, Department of Earth Sciences, Earth and Climate Cluster, VU University, De Boelelaan 1085, 1081 HV Amsterdam, The Netherlands; 10 GFZ German Research Centre for Geosciences, Telegrafenberg, 14473 Potsdam, Germany

**Keywords:** Arctic, Carbon cycle, Ocean, Permafrost, Sea ice, Tundra

## Abstract

The current downturn of the arctic cryosphere, such as the strong loss of sea ice, melting of ice sheets and glaciers, and permafrost thaw, affects the marine and terrestrial carbon cycles in numerous interconnected ways. Nonetheless, processes in the ocean and on land have been too often considered in isolation while it has become increasingly clear that the two environments are strongly connected: Sea ice decline is one of the main causes of the rapid warming of the Arctic, and the flow of carbon from rivers into the Arctic Ocean affects marine processes and the air–sea exchange of CO_2_. This review, therefore, provides an overview of the current state of knowledge of the arctic terrestrial and marine carbon cycle, connections in between, and how this complex system is affected by climate change and a declining cryosphere. Ultimately, better knowledge of biogeochemical processes combined with improved model representations of ocean–land interactions are essential to accurately predict the development of arctic ecosystems and associated climate feedbacks.

## Introduction

**Fig. 1:**
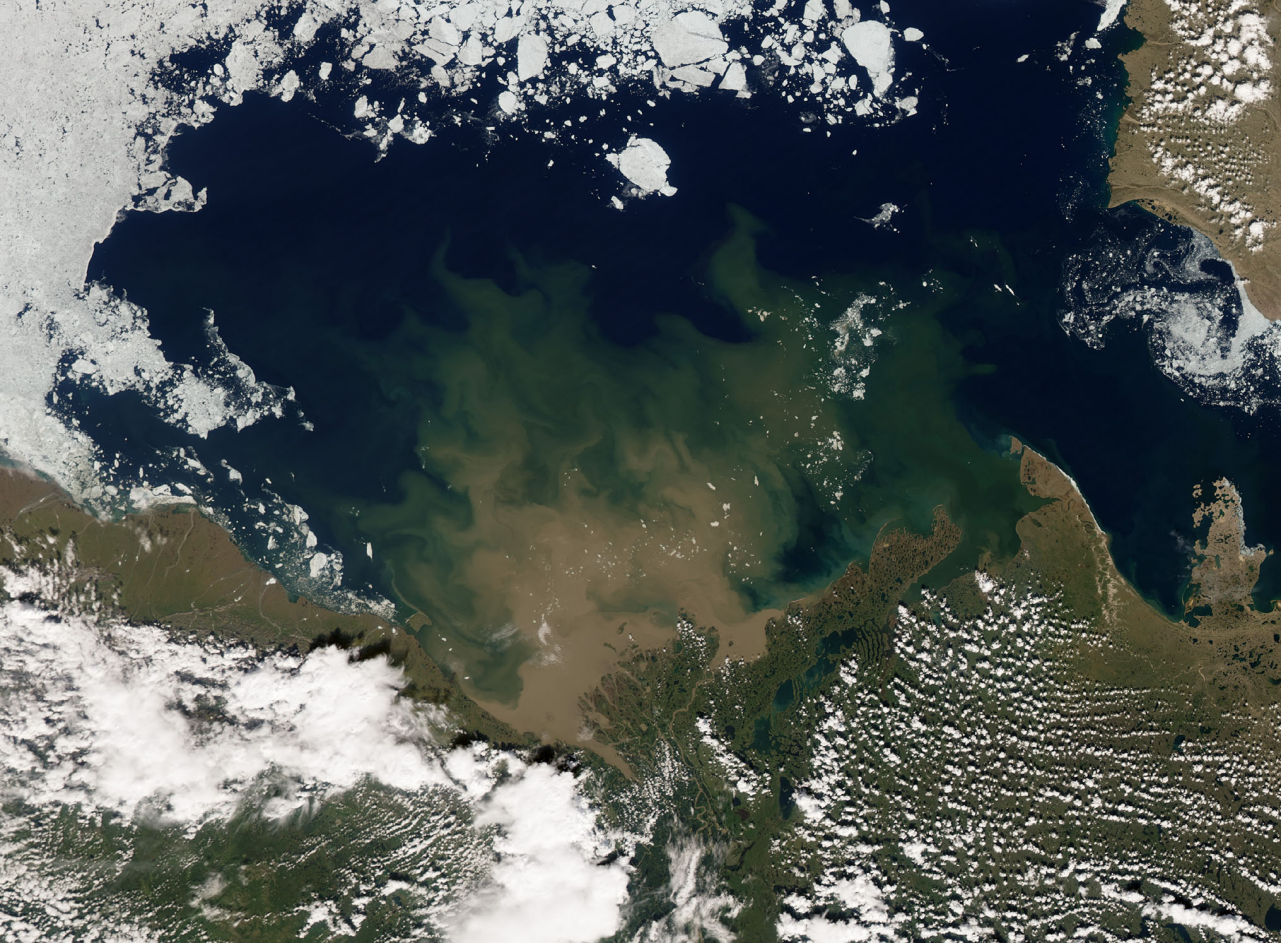
Satellite photo showing the Beaufort Sea and the Mackenzie delta. Vast sediment flows can be seen entering the ocean, containing large amounts of carbon from the terrestrial environment. Image acquired on July 5th 2012 by the Moderate Resolution Imaging Spectroradiometer (MODIS) onboard NASA’s Aqua satellite

From the perspective of an astronaut, looking down on the Earth high above the North Pole, it is self-evident that the marine and terrestrial carbon cycles of the Arctic cannot be considered separately. Huge rivers empty into the Arctic Ocean, carrying vast amounts of sediment that can be seen from space as immense swirls in the coastal region (Fig. 1). On top of the ocean floats a thin layer of sea ice that strongly governs conditions for primary production, air–sea exchange of greenhouse gases and the surface energy balance. Critically, the dramatic decline of this part of the cryosphere, exceeding even aggressive projections, is one of the main drivers for the rapidly rising temperatures in the Arctic (Screen et al. 2012), and this extends across the ocean–land boundary: About 80% of lowland tundra lies within 100 km inland from the arctic coastline. In turn, the terrestrial environment is strongly affected by the amplified warming in the Arctic: enhanced plant growth may increase carbon uptake (Bhatt et al. 2014), while permafrost thaw may lead to the release of CO_2_ and methane (Parmentier et al. 2015; Schuur et al. 2015). Permafrost degradation and enhanced rainfall might also contribute to a change in the outflow of organic and inorganic carbon (OC/IC) towards the Arctic Ocean (Vonk and Gustafsson 2013). However, the feedbacks to climate from a changing cryosphere are complex, vary over space and time, and are generally poorly understood. This article seeks to provide a comprehensive review of recent information on ecosystem–atmosphere interactions in the Arctic, carbon cycling in terrestrial and marine ecosystems of the high latitudes, and how they interact with each other in the context of sea ice decline and permafrost thaw.

Although this review focuses primarily on the carbon cycle of the northern latitudes as a whole, we will also—in light of this special issue—point out unique contributions of the Greenland Ecosystem Monitoring Programme to our understanding of the arctic carbon cycle, and the implications of the issues described above. Established in 1994, this programme is ideally suited to take on research questions that involve both the marine and terrestrial environment since the monitoring program covers a comprehensive list of parameters measured in the same areas and seasons covering the atmosphere–cryosphere–land–lake–rivers–fjord–ocean compartments at Zackenberg/Daneborg in northeast Greenland and Nuuk in southwest Greenland. Recently, arctic stations at Disko in western Greenland and Sermilik in eastern Greenland have been included in this programme. The monitoring of the carbon cycle at these Greenlandic stations, spanning the entire transition from land to river to fjord to ocean, has led to ground-breaking discoveries that have changed our perception of the functioning of the arctic carbon cycle.

## Marine carbon cycling

### Carbon exchange between water and atmosphere

Changes in arctic sea ice cover, the marine ecosystem, and the hydrological cycle could significantly affect the amount of CO_2_ that is absorbed from the atmosphere by the Arctic Ocean (typically defined as the ocean waters North of the Arctic circle). The controls on this “carbon sink” can be broadly categorized into the biogeochemical processes that determine the concentration of dissolved CO_2_ in seawater (i.e., *p*CO_2sw_), and the physical processes that determine the rate of gas exchange across the air–sea interface (i.e., the gas transfer velocity, *k*). As we will discuss in the following, both *p*CO_2sw_ and gas transfer velocity are susceptible to climate change, and therefore the Arctic Ocean carbon sink as well.

**Fig. 2:**
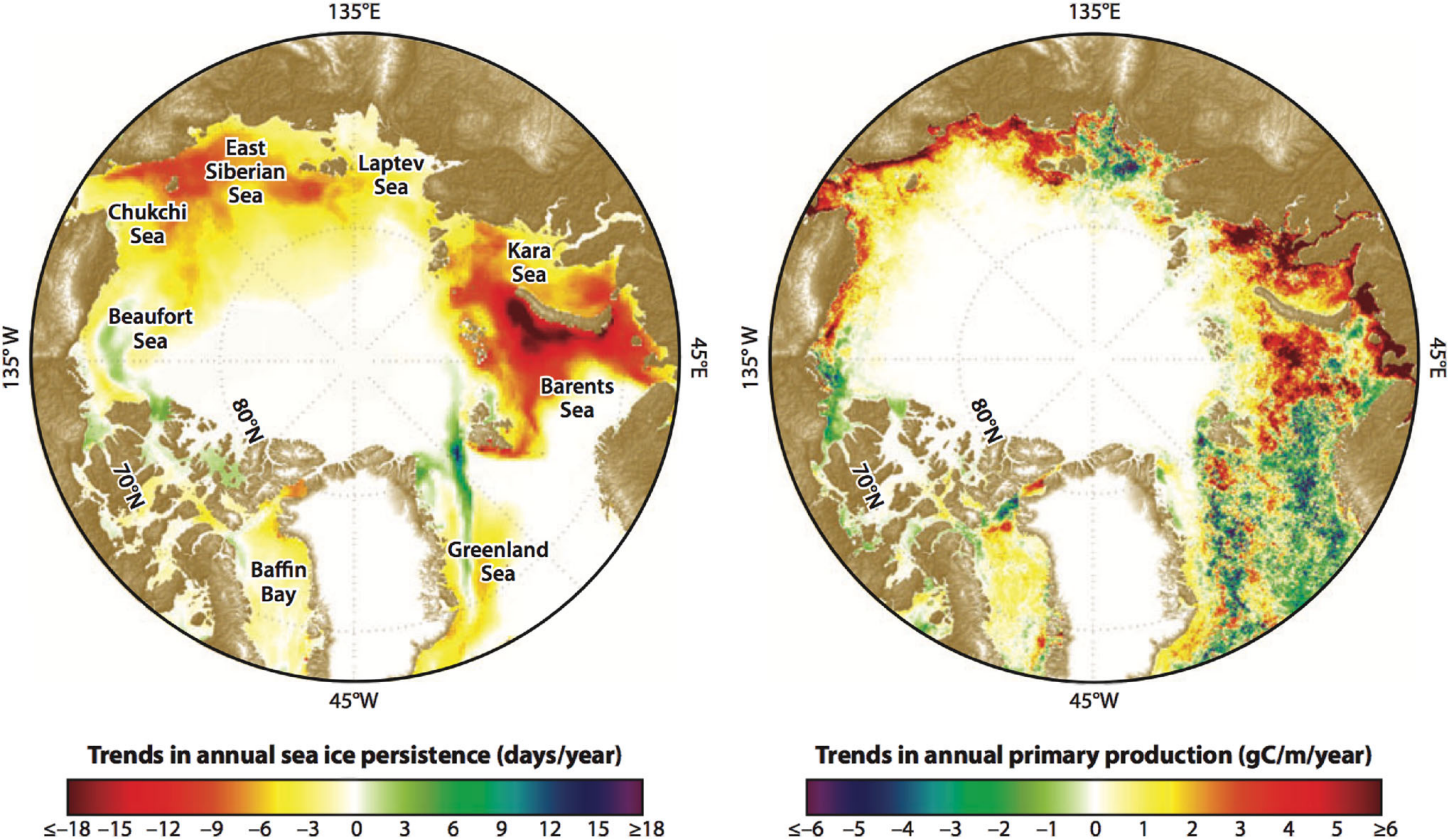
Trends in annual sea ice persistence and total annual net primary production across the Arctic Ocean and its adjacent shelf seas from 1998 to 2009 (in g C m^−2^). Primary production estimates are from Arrigo et al. (2010). Figure appeared earlier in Bhatt et al. (2014)

One of the most important biogeochemical processes for removing inorganic carbon from surface seawaters and reducing *p*CO_2sw_ is primary production. High calculated sinks for atmospheric CO_2_ in the fjords and shelf waters around southern Greenland are the result of high primary production and release of meltwater from glaciers leading to low *p*CO_2sw_ (Rysgaard et al. 2012). Increased open water and increased primary production should drive a lower *p*CO_2sw_ and increase CO_2_ uptake. Satellite observations, for example, have suggested significant Arctic Ocean production increases in response to longer growing seasons associated with sea ice loss (Arrigo and van Dijken 2011), as shown in Fig. 2. Up to this point, however, sea ice loss has occurred primarily over nutrient-rich shelf seas, while observations in the deep basin show low CO_2_ uptake capacities as a result of stratification and nutrient limitation (Cai et al. 2010; Else et al. 2013).

Nutrient limitation may become more widespread in a changed Arctic Ocean as freshening from increased river runoff and ice melt (Morison et al. 2012) suppresses vertical mixing (Nummelin et al. 2016) and reduce light availability due to increased turbidity. This could potentially lead to reduced primary production in surface waters. Primary producers on the sea floor including both micro- and macroalgae will be less limited by nutrients and are thus expected to respond directly to light availability (Glud et al. 2009; Krause-Jensen et al. 2012). Primary production associated with the sea floor may in general be an overlooked carbon pathway in the Arctic with the contribution of microalgae alone estimated to amount to 15–30% of the annual pelagic production in the Arctic (Glud et al. 2009; Attard et al. 2016). Other changes opposing production-driven *p*CO_2sw_ drawdown include increasing sea surface temperatures (Steele et al. 2008), potential increases in dissolved and particulate OC and IC fluxes of rivers (Tank et al. 2012a), and coastal erosion (Vonk et al. 2012).

Clearly, enhanced focus on responses towards reduced sea ice cover and enhanced run-off in the coastal Arctic is required to assess the changes in net productivity given projected climate change in the region. Moreover, current estimates of arctic primary productivity could be underestimated since significant phytoplankton blooms have recently been discovered below arctic sea-ice, and these are poorly represented in traditional remote sensing data (Arrigo et al. 2010). In addition, several recent studies have reported on the wide-spread occurrence of sea-ice algae aggregates below melting summer ice in the central Arctic and the Fram Strait (Boetius et al. 2013; Glud et al. 2014). It remains unclear to what extent recent reports on ice-algae aggregates reflect an actual increase in their numbers, potentially related to increased melt pond coverage (Palmer et al. 2014), or that these aggregates simply have been overlooked previously.

The importance of primary production as a sink for atmospheric CO_2_ ultimately depends on the fraction of the material that is retained in the sediment record. Marine settings generally express a close relationship between the sedimentation rate and the burial rate of organic material (Canfield 1994). This was also confirmed recently for coastal settings in the Arctic and available data suggest a relation between long-term burial of organic carbon, the pelagic productivity, and the extent of the ice-free period in arctic fjords (Sørensen et al. 2015).

In addition to changing primary productivity, the ongoing changes to arctic sea ice are also expected to permit more gas exchange across the ocean–atmosphere interface (Barber et al. 2015). Most notably, longer open water seasons will increase the amount of time that seawater is in direct contact with the atmosphere. Since gas transfer rates are much higher through open water than through the sea ice itself (Loose et al. 2014), a reduction in sea ice extent must lead to increasing mean annual gas transfer (Sejr et al. 2011; Barber et al. 2015). Since gas transfer in the open ocean is strongly driven by waves (e.g., Wanninkhof et al. 2009), the mean transfer rates may also become greater as wave generation increases in the Arctic (Asplin et al. 2014) and in ice-affected areas as the extent of the marginal ice zone changes (Strong and Rigor 2013). Increasing sea ice drift (Spreen et al. 2011) results in more lead and polynya activity, which may be potentially important to gas exchange in the Arctic (Else et al. 2011; Loose et al. 2014). However, it should be noted that not all investigations have confirmed enhanced gas transfer in such environments (Rutgers van der Loeff et al. 2014). Observations have also shown that gases can be transported through warm, thin first-year sea ice (Loose et al. 2011), potentially extending the seasons and locations involved in air–sea gas exchange. Current estimates of diffusion rates through ice suggest that this exchange may be insignificant compared to direct air–sea exchange (Loose et al. 2011; Crabeck et al. 2014a), but we lack observations during the more dynamic spring break-up and fall freeze-up periods.

### Sea ice interactions with carbon cycling

**Fig. 3:**
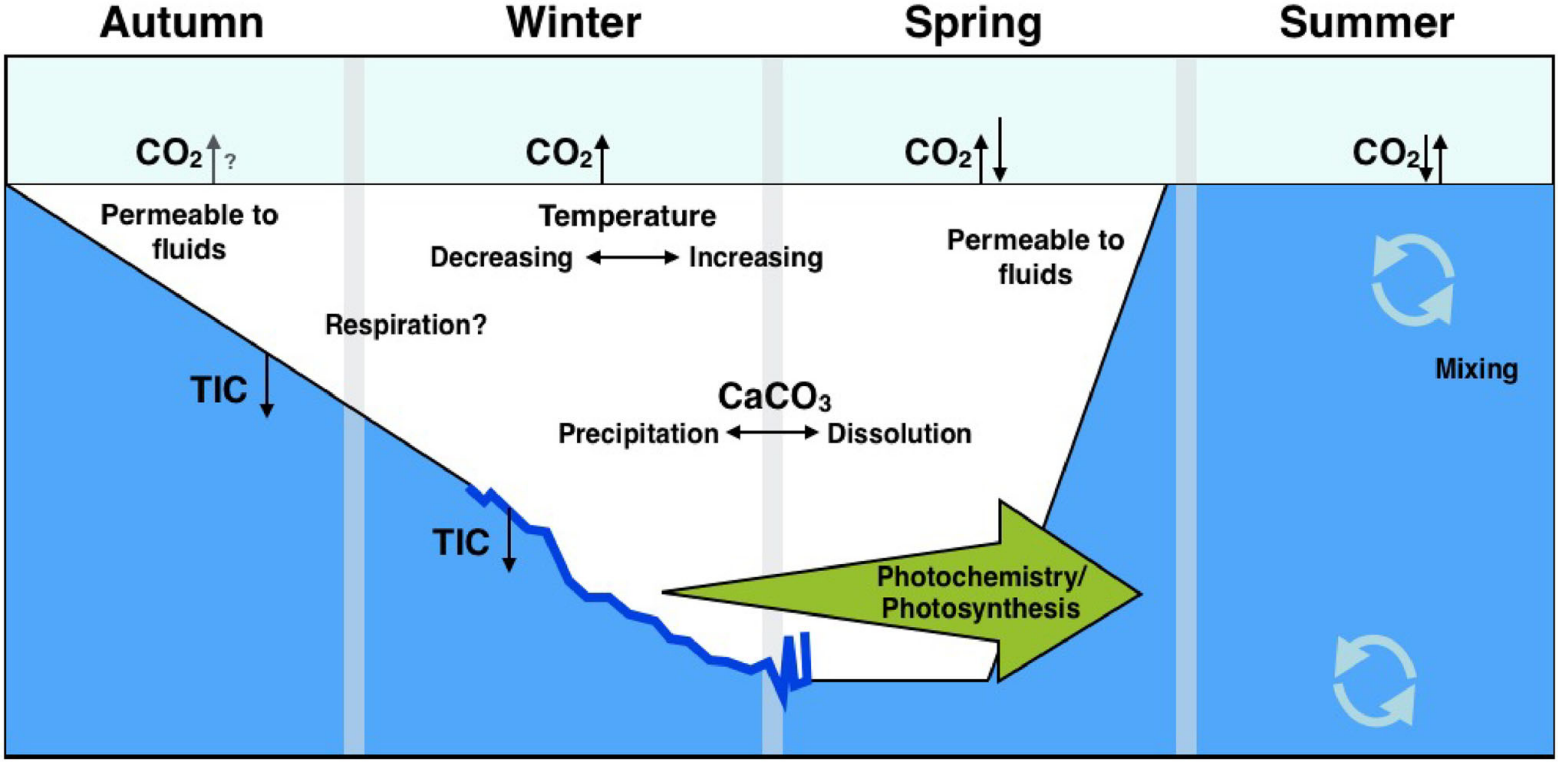
Summary of the various carbon cycling processes in the ocean related to sea ice. In autumn, carbon is rejected together with brine during sea ice formation, which sinks because of its high density (*TIC* total inorganic carbon). The permeability of the ice is determined by temperature, and the ice–air exchange of CO_2_ is governed by the difference in partial pressure of CO_2_ (*p*CO_2sw_) with the atmosphere. When sea ice melts, ikaite crystals within the ice dissolve and alter the alkalinity of surface waters, lowering *p*CO_2sw_, and stimulating uptake. Furthermore, if the ice is thin enough, sunlight can penetrate and stimulate photosynthesis. In areas without sea ice, the exchange with the atmosphere is determined by the *p*CO_2sw_ difference between the air and the ocean surface. Adapted from Miller et al. (2011)

While an increase in open water due to sea ice decline clearly affects the arctic carbon sink in one way or the other, traditionally the role of the ice-covered part of the ocean has been largely ignored since sea ice was assumed to impede gaseous exchange with the atmosphere (Tison et al. 2002). However, sea ice itself is permeable above approximately −5 °C (e.g., Golden et al. 1998) and can support gas exchanges, as shown from observations across the Arctic—including Young Sound, northeast Greenland (Miller et al. 2011; Geilfus et al. 2013; Sievers et al. 2015). In addition, recent studies have shown that physical and chemical processes in the sea ice itself may act as an important control on *p*CO_2sw_ levels of the sea surface (see e.g., Rysgaard et al. 2009; Parmentier et al. 2013; Delille et al. 2014), which also followed from fieldwork in Young Sound, and from sampling along the northeast Greenlandic coast (Rysgaard et al. 2007, 2009). During sea ice growth, the precipitation of the carbonate crystal, ikaite, increases the CO_2_ concentration in the brine. If the major part of this CO_2_ is rejected via brine drainage and mixed into the interior of the ocean while the ikaite crystals remain trapped in the sea ice matrix, then the release of the alkalinity to the surface oceans by the dissolution of ikaite enhances the air–sea CO_2_ uptake during ice melt. Preliminary budget calculations of the potential size of the CO_2_ flux related to this sea ice pump show an uptake of 14–31 Tg year^−1^ in the Arctic and 19–52 Tg year^−1^ in the Antarctic (Rysgaard et al. 2011; Delille et al. 2014). This process has been suggested to be an important mechanism contributing to the ocean CO_2_ sink, not only today but also during the Last Glacial Maximum (Bouttes et al. 2010). A schematic overview of sea ice-related fluxes is shown in Fig. 3.

Methane emissions have also recently received attention in the sea ice zone since several studies indicated significant sources of methane in the Arctic Ocean—up to 17 Tg CH_4_ year^−1^ (Damm et al. 2010; Shakhova et al. 2010, 2014; Kort et al. 2012; Vancoppenolle et al. 2013). Gas hydrates represent a large potential source of methane from the ocean floor (Kretschmer et al. 2015), and may be vulnerable to climate change. Although gas plumes have been reported to occur extensively off the coast of Spitsbergen, it appears that the water column in this location is deep enough to act as an efficient filter, and little methane reaches the atmosphere (Lund Myhre et al. 2016). Other atmospheric measurements conducted near the Laptev Sea indicate that previous bottom-up estimates have strongly overestimated the importance of the Arctic Ocean as a methane source, perhaps by a factor of 4 or 5 (Berchet et al. 2016; Thornton et al. 2016). In that same region, it has been shown that methane released upon the degradation of subsea permafrost is quickly oxidized in the overlying sediment, limiting the potential for large increases of methane to reach the water column (Overduin et al. 2015). Model studies also indicate that gas hydrates respond slowly to climate change, since warming at the sea surface, e.g., due to sea ice decline, takes a long time to penetrate to depths where gas hydrates are located (Parmentier et al. 2013; Kretschmer et al. 2015). Taken together, it is possible that the Arctic Ocean may not be the fast-changing or large source of methane as previously feared (Shakhova et al. 2010, 2014).

However, many uncertainties remain and sea ice has also been suggested to regulate methane levels in the Arctic Ocean through two other mechanisms: shielding of methane emissions from the ocean, and consumption of methane (He et al. 2013). Research near Nuuk, southeast Greenland, suggests that river runoff from land may be an important methane source in sea ice in coastal areas and that sea ice can be a sink for CO_2_ while being a source for methane (Crabeck et al. 2014b).

### Budgets and trends

In general, the marine Arctic is considered to be a carbon sink and several studies agree on an overall uptake of about 0.1–0.2 Pg C year^−1^. However, this overall agreement has been obtained despite large regional differences of estimated transports and air–sea CO_2_ exchanges in the marine Arctic; Jeansson et al. (2011) estimated an uptake of 0.2 Pg C year^−1^ in the Nordic Seas and Schuster et al. (2013) estimated, correspondingly, an uptake of 0.12 and 0.21 Pg C year^−1^ for the Arctic Ocean (>76°N) and North subpolar North Atlantic (49°N—76°N), respectively. Arrigo et al. (2010) used satellite data to estimate an uptake of 0.12 Pg C year^−1^ for an area covering most of the marine Arctic and MacGilchrist et al. (2014) estimated a total air–sea uptake of 0.17 Pg C year^−1^ for the area. Model studies of the Arctic Ocean have resulted in an uptake of 0.05 Pg C year^−1^ (McGuire et al. 2009), whereas Manizza et al. (2013) estimated an uptake of 0.06 Pg C year^−1^ for the entire marine Arctic. Thus, further observational and regional model studies of the marine Arctic are required to reduce the uncertainty among current estimates.

Following from the large uncertainties in the functioning of the Arctic Ocean carbon sink, we lack robust predictions of how the uptake of carbon may evolve in the future. Attempts to quantify past changes using biogeochemical models have suggested an increasing sink of 0.9 Tg C year^−1^ (Schuster et al. 2013) to 1.4 Tg C year^−1^ (Manizza et al. 2013). These estimates are in line with Jutterstrom and Anderson (2010), who predicted an increase in uptake of 1.3 Tg C year^−1^ based simply on increased exposure of the surface ocean to the atmosphere. Whether or not this rate of increase can be sustained long-term remains unclear due to our incomplete understanding of the biogeochemical and physical processes controlling air–sea CO_2_ exchange in the Arctic.

## Arctic terrestrial carbon cycling

### Size and characteristics of the terrestrial arctic soil carbon reservoir

**Fig. 4:**
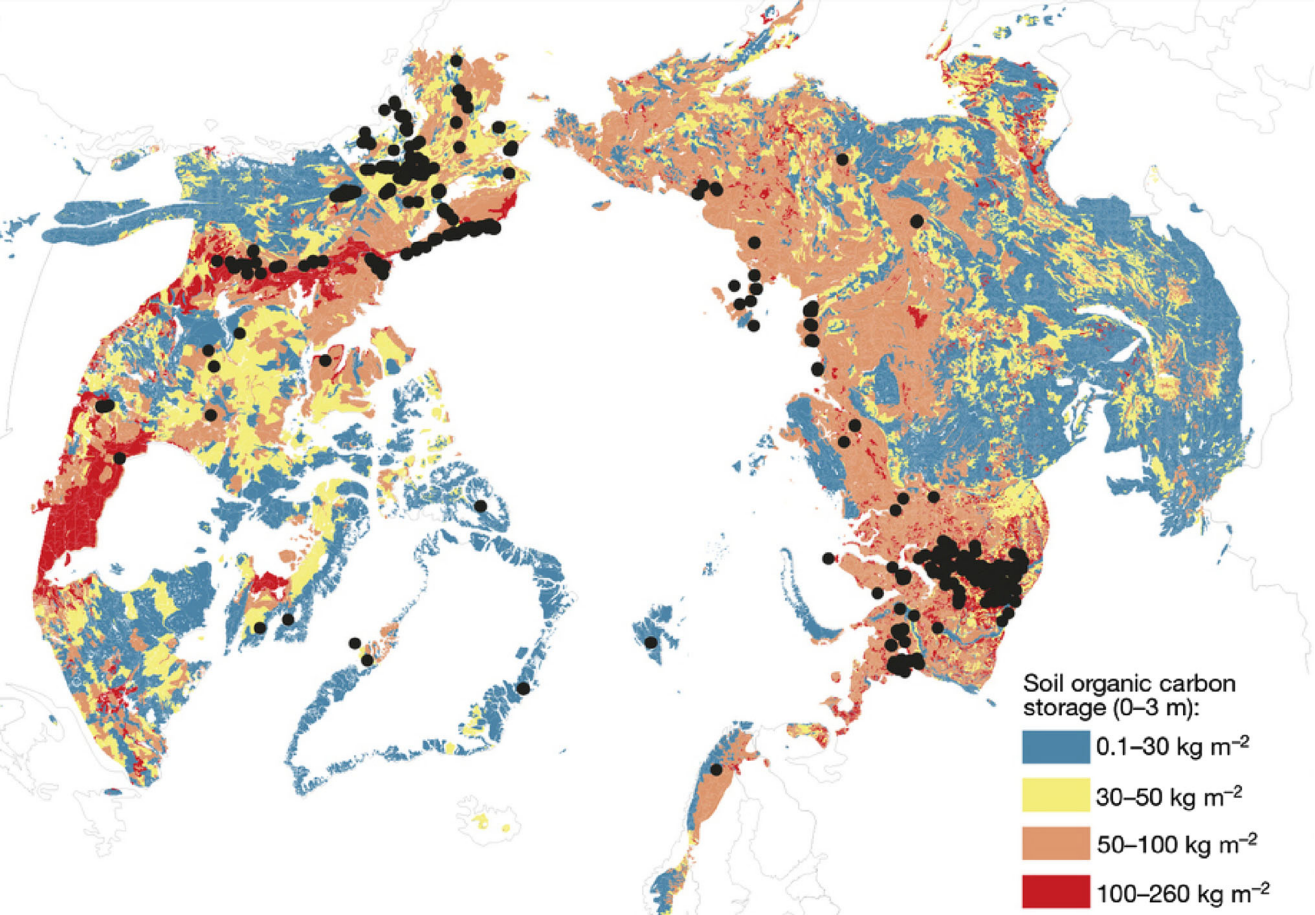
Soil organic carbon pool (kg C m^−2^) contained in the 0–3 m depth interval of the northern circumpolar permafrost zone. *Points* show field site locations for 0–3 m depth carbon inventory measurements; field sites with 1 m carbon inventory measurements number in the thousands and are too numerous to show. Adapted from Hugelius et al. (2014)

Low temperatures and wet conditions prevail in landscapes across the Arctic, and these conditions favor low decomposition rates, and the accumulation and preservation of organic matter. Over the course of millennia, vast amounts of carbon have built up in arctic soils, especially in areas of permafrost (soil that is frozen for at least two consecutive years)—which cover about 25% of the land area in the northern hemisphere. The most recent estimates for arctic soil carbon stocks, as shown in Table 1, converge on a range between 1400 and 1850 Pg C for all northern permafrost soils (750–1024 Pg C for the top 3 m, 400–407 Pg C for yedoma and 241–250 Pg C for alluvial deposits). However, the uncertainties associated with these estimates are large, and following the analysis of a significantly larger database, including new sampling locations from Greenland and across the Arctic (Fig. 4), a revised estimate arrived at lower estimates for yedoma (181 ± 54 Pg C) and alluvial deposits (91 ± 52 Pg C) in particular (Hugelius et al. 2014). Still, vast stocks of organic carbon are contained in arctic soils and amount to about 50% of the world’s global soil carbon (Tarnocai et al. 2009; Hugelius et al. 2014). The emission of CO_2_ or methane following decomposition of this carbon is a potentially important feedback to climate warming.

**Table 1:** Recent estimates of soil carbon in the northern circumpolar permafrost zone

Source	0–100 cm, Pg C	0–300 cm, Pg C	>300 cm(yedoma), Pg C	>300 cm (delta/alluvial), Pg C	Total, Pg C
Tarnocai et al. (2009)	496	1024	407	241	1672
Schuur et al. (2008), McGuire et al. (2009)	Not determined	750	400	250	1400–1850
Hugelius et al. (2014)	472 ± 27	1035 ± 150	181 ± 54	91 ± 52	1307 ± 170

The rate at which soil carbon can be transferred to the atmosphere depends, among other things, on the decomposability (lability) of soil organic matter (SOM), which shows considerable variability. Aerobic incubation of organic and mineral soil cores—collected from Zackenberg, northeast Greenland, as well as Alaska and northern Siberia—showed that the fraction of SOM that turns over in less than a year (‘fast pool’) was less than 5% for all soils (Schädel et al. 2014). The ‘slow pool’ (defined here by a turnover time of 5–15 years) varied between organic and mineral soils, of which organic soils showed the highest values and highest variability. A comparable study with anaerobic incubations showed differences in the anaerobic CO_2_:CH_4_ production ratio (lowest for tundra sites), and in overall anaerobic CO_2_ and methane production (greatest for organic soils and inundated soils, and least for deeper horizons). Methane production was more than four times greater in soils from graminoid (grass) and shrub-dominated sites than in soils from forested sites, indicating that the vegetation community can influence methane fluxes considerably, as shown for example by field observations in Zackenberg (Ström et al. 2003). Between aerobic and anaerobic incubations, however, it appears that potential carbon emissions are largest, and dominated by CO_2_, under aerobic conditions—even when accounting for the larger global warming potential of methane (Schädel et al. 2016).

Despite incubation studies showing a clear potential for permafrost soils to release substantial amounts of greenhouse gases, the extent to which carbon may be available for decomposition under natural conditions is dependent on its conservation in frozen ground, vulnerability to permafrost degradation, and burial depth (McGuire et al. 2010; van Huissteden and Dolman 2012). Cryoturbation (vertical movement of soil resulting from freeze–thaw processes) mixes carbon to deeper levels in the soil, thereby potentially removing it from layers of rapid decomposition (Kaiser et al. 2007). On the other hand, permafrost thaw may cause degradation processes and soil subsidence resulting in lake and pond formation and erosion, processes that can expose soil carbon to either anaerobic decomposition causing methane and CO_2_ emissions, aerobic decomposition resulting in CO_2_ emissions, or transport as dissolved and particulate OC to rivers and streams to lakes and the sea (van Huissteden and Dolman 2012; Vonk and Gustafsson 2013). Actual emissions from permafrost soils may therefore be quite different from the potential decomposition rates obtained in incubation studies.

### Atmospheric exchanges

Most of the direct observational studies of the exchange of CO_2_ between tundra and the atmosphere have been conducted in summer, during the growing season, when plants photosynthesize and take up CO_2_ from the atmosphere. This uptake can partially or completely offset any losses arising from the decomposition of soil carbon, and observations indicate that, throughout the Arctic, tundra has been a sink for atmospheric CO_2_ during the summer (McGuire et al. 2012). The existing observations do suggest that there are differences in net summer CO_2_ exchange among different tundra types, since no overlap exists in the confidence intervals of reported net CO_2_ uptake of wet tundra (−27 to −59 g C m^−2^ summer^−1^) when compared to dry tundra (−11 g C to 21 g C m^−2^ summer^−1^).

While, in general, net carbon uptake occurs during summer, large uncertainties exist on the amount of carbon released during the long arctic winters when plant growth has ceased but soil respiration may continue at a slow and steady pace. Only a handful of studies have estimated the exchange of CO_2_ during the cold season since the harsh conditions in that time of year complicate logistics and continued monitoring. The studies that are available indicate that tundra ecosystems are sources of CO_2_ to the atmosphere during the cold season (McGuire et al. 2012), but given the few studies that have been conducted, it is not clear whether the strength of sources differs among subregions or tundra types. A growing number of observationally based studies are attempting to fill this knowledge gap and estimate annual CO_2_ exchange between tundra and the atmosphere (McGuire et al. 2012), but due to the large associated uncertainties there is considerable doubt on whether tundra is a small or near neutral carbon sink on an annual basis. Current estimates range from −291 to 80 Tg C year^−1^, with a central estimate of −110 Tg C year^−1^ (McGuire et al. 2012).

Besides CO_2_, there is an even greater uncertainty in arctic tundra methane emission estimates, primarily due to spatio-temporal variability not adequately captured by the current sparse measurement networks, and uncertainty of the extent of wetlands in the Arctic. In general, models tend to predict higher wetland emissions from the Arctic than observations. A comparison between observations and models showed that, according to the former, tundra emitted 14.7 Tg CH_4_ year^−1^ (0–29.3 Tg CH_4_ year^−1^ uncertainty range) during the 1990s and early 2000s, while models estimated ~35 Tg CH_4_ year^−1^ (21–47 Tg CH_4_ year^−1^). The lower number from observations is largely in agreement with the review of bottom-up (BU) and top-down (TD) methane emission estimates by Kirschke et al. (2013), who suggest a Eurasian boreal wetland source of 14 Tg CH_4_ year^−1^ (min–max range 9–23) and 9 (4–13) Tg CH_4_ year^−1^ for TD and BU, respectively, with a TD estimated soil sink of 3 (1–5) Tg CH_4_ year^−1^.

Besides modeling and ground-based measurements, recent developments include the increased use of airborne measurements, which have the great advantage of avoiding biases induced by logistical constraints on ground-based study site selections or problems that arise during upscaling—such as the underestimation of regions of net methane uptake (Jørgensen et al. 2014). Airborne measurements also inherently include previously often neglected sources such as freshwater systems and geologic sources, which can be significant sources of methane and CO_2_ (Walter Anthony et al. 2012; Wik et al. 2016). A study that combined aircraft concentration data with inverse modeling estimated that regional fluxes averaged over all of Alaska for the period from May to September 2012 amounted to 2.1 ± 0.5 Tg CH_4_ (Chang et al. 2014). A recent study in the same region that combined both ground-based and airborne measurements showed that methane emissions in the rest of the year, during the cold season, can be just as large, contributing ~50% to the annual budget (Zona et al. 2016). When including cold season emissions, it was suggested that total arctic methane emissions may be as high as 23 ± 8 Tg CH_4_ year^−1^ (Zona et al. 2016).

The importance of the cold season to the annual methane budget had been shown earlier in Zackenberg by Mastepanov et al. (2008). At this site, a large peak in emissions was observed during the freeze-in period, likely related to the formation of ice in the ground that lowers the pore space and raises pressure, which causes gases to be squeezed out of the ground (Pirk et al. 2015). Similar large peaks of methane have since been observed in Adventdalen, Svalbard (Pirk et al. 2017) and in Alaska (Zona et al. 2016), showing that the winter season is a dynamic period that has to be included in observations to accurately assess annual methane budgets.

## Lateral carbon flows

### Freshwater carbon transport pathways

The hydrological cycle is an important connecting factor between the Arctic Ocean and the surrounding land. Aquatic systems integrate terrestrial processes, serve as reactive transport pathways, and as locations for short- or long-term burial along the path from land to ocean (Vonk and Gustafsson 2013). This freshwater carries sediments, nutrients, and organic matter, and has a relatively large influence on the adjacent ocean: Despite holding only 1% of the global ocean volume, approximately 10% of all river discharge in the world flows into the Arctic Ocean. Combined, the eight largest arctic rivers export an estimated ~249 Tg of sediment and ~40 Tg organic carbon (OC) to the Arctic Ocean each year (Holmes et al. 2002; McGuire et al. 2009). Additionally, coastal erosion is estimated to deliver 430 Tg sediment (Rachold et al. 2004) and 5–14 Tg OC per year (Rachold et al. 2004; Vonk et al. 2012). Once these flows of carbon arrive in the ocean, they may be (further) degraded, released to the atmosphere (Anderson et al. 2009), or buried for long-term storage in sediments. All of these processes may be affected by climate change, altering the interaction between ocean and land.

To grasp future changes in lateral carbon flows, a better understanding of permafrost is paramount. About three quarters of the area draining into the Arctic Ocean is underlain by permafrost, but the hydrology of this complex environment, under increasing pressure from global warming, is poorly understood. However, the interplay between permafrost and the hydrological cycle is bound to have a considerable impact on the lateral flows of carbon in the Arctic, and ultimately the flux to the atmosphere. For example, a deepening of the active layer—the top of the soil in permafrost environments that thaws each year—may increase the drainage of water, and runoff. Alternatively, soil subsidence due to permafrost thaw may create new depressions in the landscape, increasing wetness (Lee et al. 2014). This may enhance the expansion of lakes, which are currently estimated to emit as much as 13 to 16.5 Tg CH_4_ year^−1^ North of ~50°N (Bastviken et al. 2011; Wik et al. 2016), but this process is ultimately limited by fluvial and subsurface drainage (Jones et al. 2011; van Huissteden et al. 2011). Besides, thermokarst lakes formed through permafrost thaw can evolve from being net emitters of greenhouse gases to locations of long-term sequestration of carbon, converting these lake basins to net carbon sinks (Walter Anthony et al. 2014).

Changes in lake emissions are highly uncertain due to a very limited body of reliable data on lake expansion. This requires extensive multi-year high-resolution remote sensing studies and has to take into account any non-climatic lake level changes (Jones et al. 2011). The few studies that do exist have found lakes expanding in some areas, while declining in other parts of the permafrost zone (Smith et al. 2005; Walter et al. 2006; Jones et al. 2011). These differences are most likely related to varying stages of permafrost degradation, drainage capacity of the landscape, and associated changes in hydrology. Overall, however, permafrost thaw increases hydrological connectivity within landscapes, which leads to increased groundwater input and winter base flow (Bense et al. 2009). Research has shown that arctic rivers have discharged more water into the ocean, both in Eurasia (Peterson et al. 2002) and North America (Déry et al. 2009).

Specifically for Greenland, melting of the Greenland Ice Sheet has accelerated in recent decades, and rates of annual net ice mass loss have more than doubled during the 2003–2010 period when compared to 1983–2003 (Kjeldsen et al. 2015). At present, glacial melt water from the Greenland Ice Sheet contributes 0.5 to 1.7 Tg C year^−1^ to the coastal ocean, following from microbial activity on the ice sheet surface and biogeochemical processes at the ice sheet bed (Lawson et al. 2014), and this may increase as melting accelerates. More importantly, the glacial melt water also has numerous indirect impacts on marine carbon cycling such as light and nutrient availability or an undersaturation of surface waters that influence patterns of primary production and CO_2_ conditions in surface waters (Sejr et al. 2011; Murray et al. 2015).

### Changes and trends in lateral carbon flows

Changes in the arctic hydrological cycle, e.g., enhanced precipitation or altered runoff, will likely affect the transport of organic matter and sediments from land to ocean. The presence or absence of water, snow, and ice plays an important role in determining the rate of carbon release from thawing permafrost as well as its eventual release into the atmosphere. Loss of sea ice, for example, has increasingly exposed the arctic coastline to storm and wave impacts, which together with warmer air and water temperatures enhances carbon fluxes from the land to the ocean due to coastal erosion (Jones et al. 2009). When considering all land that drains into the Arctic Ocean, however, it remains unclear whether the net OC export will increase or decrease due to climate change. This is partly due to the lack of long-term time series, but also due to region- and landscape-specific responses of OC export to permafrost thaw (Tank et al. 2012b). The Yukon River, for example, showed a 40% decrease in discharge-normalized dissolved OC export from 1978 to 2003—likely attributed to increasing hydrological flow paths and intensified processing of OC within soils (Striegl et al. 2005). On the other hand, dissolved OC concentrations are significantly higher in permafrost-free versus permafrost-dominated sub-watersheds in west Siberian peatlands (Frey et al. 2007) and in small watersheds in interior Alaska (MacLean et al. 1999), suggesting that dissolved OC export will increase when permafrost thaws at these locations.

The degradation potential of OC released from thawing permafrost is also an important factor: Carbon released during river base flow appears more labile than summer carbon fluxes (Wickland et al. 2012) and old permafrost carbon is more labile than surface soil carbon (Vonk et al. 2013). The fluxes of both types of labile carbon (base flow carbon, old permafrost carbon) will likely increase in a warming climate, potentially leading to increased conversion of aquatic carbon to greenhouse gases.

In Greenland, large differences exist in the influence of terrestrial carbon on the marine system, depending on the input of glacial meltwater and the ratio between autochthonous and allochthonous carbon. In the highly productive fjords near Nuuk in southwest Greenland, the relative importance of terrestrial carbon is low (Sejr et al. 2014), while the influence of the land is much higher in the often sea ice-covered, and low productive, Young Sound of northeast Greenland. In that system, 40% of particulate organic material in the sediment is of terrestrial origin (Rysgaard and Sejr 2007). Large uncertainties still exist if and where carbon transported by freshwater systems will be emitted to the atmosphere, or whether it is buried in sediment during transport towards the open ocean (Vonk and Gustafsson. 2013).

## Integration of the arctic carbon cycle and consequences for future projections

### Implications of sea ice decline for the terrestrial Arctic

Besides lateral flows of carbon that can affect the ocean, the reverse is also true: the ocean influences the terrestrial Arctic, most importantly through the massive decline in sea ice during recent decades. Loss of sea ice has exposed more open ocean water with a lower albedo, resulting in increased absorption of solar radiation (Pistone et al. 2014). Less ice and more absorbed energy lead to higher air temperatures, and sea ice decline may be responsible for as much as 50 to 75% of near-surface warming in the Arctic, especially in the autumn (Screen et al. 2012). Moreover, precipitation may also increase as a result of the disappearing sea ice (Bintanja and Selten 2014). This strong impact on the arctic climate from sea ice loss is expected to affect emissions since both temperature and wetness strongly control the terrestrial carbon cycle. It is therefore important to understand how sea ice decline influences the terrestrial carbon cycle, to improve forecasts of change. Moreover, the dissimilar warming from sea ice decline throughout the year may lead to varying responses in respiration, photosynthesis, and methane production that currently may be underappreciated.

**Fig. 5:**
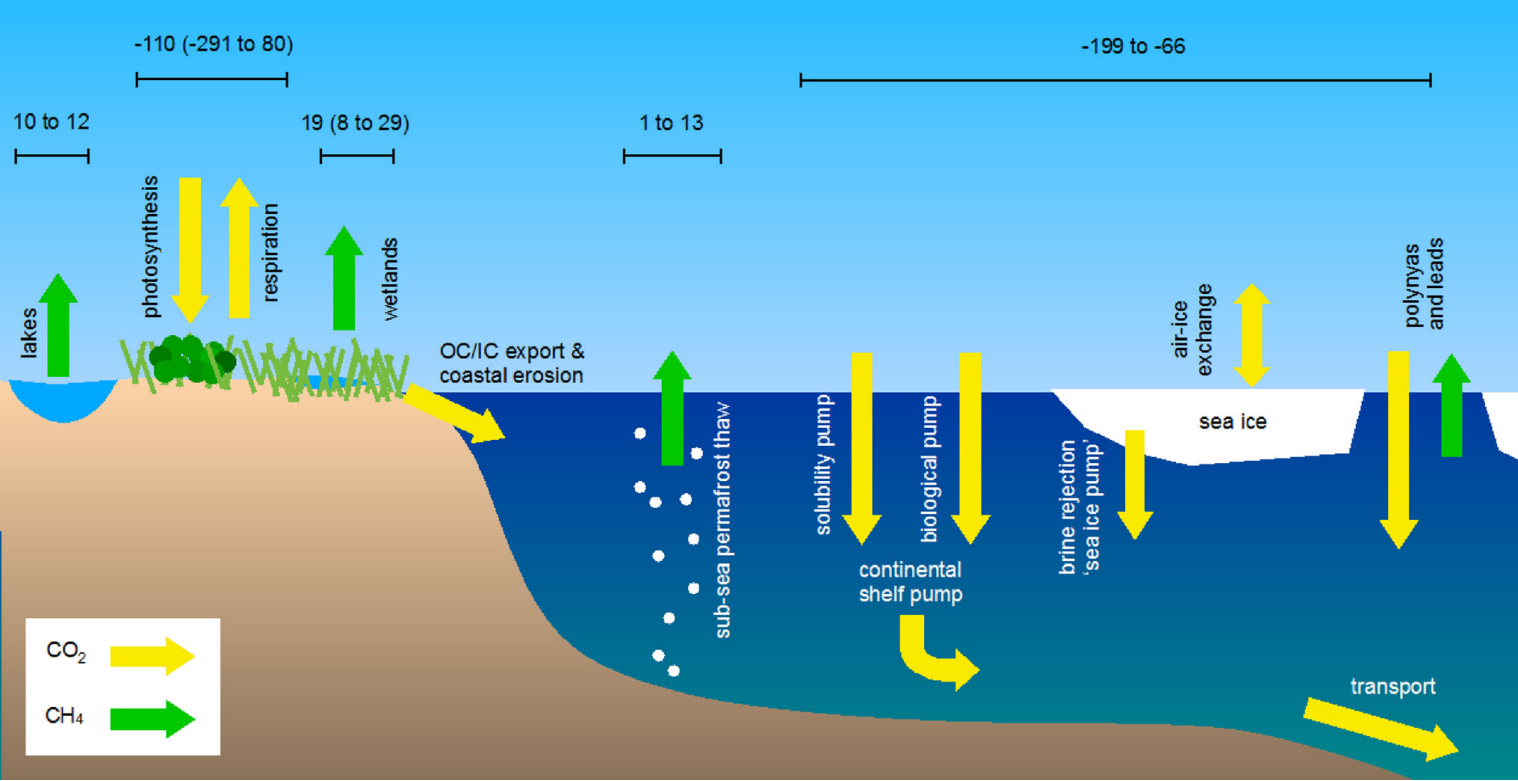
Simplified representation of arctic carbon fluxes that are possibly influenced by sea ice decline and permafrost thaw. On land, plants take up carbon while microorganisms in the soil produce methane and respire CO_2_. Lakes are net emitters of methane, and organic and inorganic carbon (dissolved and particulate) flow into the ocean through freshwater systems. In the ocean, methane can be released from thawing subsea permafrost, and CO_2_ is absorbed due to an undersaturation of CO_2_ in the water compared with the atmosphere. In addition, multiple fluxes are closely associated to sea ice. Current best estimates of atmospheric fluxes are given in Tg C year ^−1^, where available. Note that the emission estimate for lakes is for the area North of ~50º N rather than the narrower definition of arctic tundra for the other terrestrial fluxes. When available, uncertainty ranges are shown in brackets. The arrows do not represent the size of each flux. Adapted from Parmentier et al. (2013)

A connection between sea ice decline and changes in terrestrial greenhouse gas exchange appears likely, and may act on many different processes as shown in Fig. 5. However, the magnitude of this impact is unclear (Parmentier et al. 2013; Bhatt et al. 2014) due to varying complicating factors. The net carbon uptake of the Arctic, for example, is defined as the difference of two large opposing fluxes: photosynthesis and respiration. This difference is small in comparison, leading to high interannual variability. Long time series are therefore needed to understand the direction of change, such as a 20-year warming study in Alaska that found that increased plant productivity and soil litter input was offset by greater soil respiration (Sistla et al. 2013). As a result, the amount of carbon stored in above-ground biomass rose, but the net carbon balance in the soil was near zero. Plant responses to increasing temperatures vary across the Arctic, however, with a low climate sensitivity in Greenland (Myers-Smith et al. 2015). Indeed, an analysis of an 11-year dataset of carbon exchange in Zackenberg showed that higher temperatures did not stimulate photosynthesis in the long term, while still raising respiration (Lund et al. 2012). Higher temperatures can therefore, depending on the response of the vegetation, also reduce net carbon uptake or turn the ecosystem into a source in some parts of the Arctic. Moreover, the highest temperature increases related to sea ice decline occur in the autumn, when photosynthesis has ceased but soil respiration and methane emissions continue. Sea ice decline may, therefore, lead to a larger release of greenhouse gases especially during that time of year (Parmentier et al. 2015).

Besides an altered uptake of carbon, large consequences for the permafrost itself can arise from changes in the vegetation structure of tundra. Shrubs shield the ground from solar radiation, effectively cooling the ground (Blok et al. 2010). An expansion of shrubs could therefore locally counteract permafrost thaw. However, the reverse is also true: a removal of shrubs leads to permafrost collapse, wetter conditions and an increase in methane emissions (Nauta et al. 2014). The way in which arctic shrubs respond to sea ice decline and associated warming is thus of high importance to the stability of permafrost soils and the release of its carbon stores in the form of greenhouse gases.

Another important control on the stability of permafrost soils is snow depth. A thick snow pack in winter insolates the ground from the coldest temperatures, effectively raising annual ground temperature. A simple snow manipulation experiment in Sub-Arctic Sweden showed that a doubling of the snow depth led to permafrost degradation and vegetation change in just a few years (Johansson et al. 2013). Since sea ice decline is expected to not only raise temperatures, but also precipitation (Bintanja and Selten 2014), it is important to assess how these climatic changes lead to a change in vegetation structure, snow distribution, and ultimately permafrost stability.

Although model simulations indicate that sea ice-induced warming increased arctic methane emissions by 1.7 Tg CH_4_ year^−1^ in 2005–2010, when compared to the 1980s (Parmentier et al. 2015), empirical verification thereof in the field is complicated by a scarcity of observations. Then again, a large-scale analysis of measurements from 303 different sites, including Zackenberg, showed that methane emissions in the Arctic are mostly controlled by temperature and depth of the water table (Olefeldt et al. 2013). If sea ice decline leads to higher temperatures and concurrently the Arctic does not become drier, then methane emissions are expected to increase. However, large regional differences are still to be expected (Watts et al. 2014). For example, an analysis of three decades of atmospheric measurements in Barrow, Alaska, showed that methane emissions had not increased despite increasing temperatures in the region (Sweeney et al. 2016), which may be related to a regional drying trend (Liljedahl et al. 2016). A significant increase was shown for November–December, however, which was attributed to increases in late-season emissions. This fits with model simulations that predict strongest methane emission increases in autumn due to sea ice decline (Parmentier et al. 2015). In addition, autumnal warming may also increase CO_2_ emissions from tundra due to higher respiration (Webb et al. 2016). However, it remains unclear whether these higher autumn emissions are larger than gains in carbon from enhanced plant growth in summer. Continued monitoring of the fall and early winter period is therefore essential to assess the impact of sea ice decline and a warming Arctic on the permafrost carbon feedback.

### Modeling of the integrated arctic carbon cycle

Studies using earth system models (ESMs) tend to acknowledge the warming impact of sea ice decline on the terrestrial environment, but often omit the extra step of assessing the consequences for the terrestrial carbon cycle (Lawrence et al. 2008; Screen et al. 2012)—despite the many connections and potential for change as outlined in this review. Besides, ESMs tend to ignore lateral flows from the terrestrial to the marine environment (Anav et al. 2013; Burd et al. 2016), even though it has long been known that the inclusion of riverine carbon input can have a significant impact on the ocean (Aumont et al. 2001). This input may be especially significant for the Arctic Ocean due to its small size compared to the relatively large riverine inflow. However, accurate simulation of small-scale coastal processes is often complicated by the large grid cell size of these models. Also, ESMs tend to vary wildly in their representation of permafrost (Koven et al. 2012), which signifies the long way that ESMs still have to go to reliable simulate the full dynamics of the arctic carbon cycle, including land–ocean transport.

Rather than all-encompassing ESM simulations, regionally applied models may be more effective at representing the interaction between ocean and land for the moment. For example, by coupling dissolved OC export from a terrestrial biosphere model to an ocean model, these two parts of the carbon cycle can interact. Indeed, a modeling effort focusing on the Arctic basin showed that changes in dissolved OC become significant at the decadal scale (McGuire et al. 2010). Terrestrial biosphere models can also be applied to identify links to sea ice decline either dynamically through coupling to regional climate models, to evaluate processes at a higher resolution (see e.g., Zhang et al. 2014), or to identify regional connections through offline forcing with reanalysis products (Parmentier et al. 2015). Such analyses can be valuable to identify the functioning of these links, and by what magnitude an amplified warming induced by the retreat of sea ice is affecting the carbon cycle. Moreover, changes in terrestrial ecosystems may affect sea ice decline in return—at least in the long term. Jeong et al. (2014) showed—under a doubling of atmospheric CO_2_—that the predicted expansion of vegetation in the Arctic lowers surface albedo, leading to additional warming of the atmosphere, and ultimately more sea ice melt.

These, and other, regionally applied modeling studies have shown that sea ice, the atmosphere, and the adjacent land are intricately connected and cannot be considered in isolation. Development of ESMs should, therefore, include a focus on improving the connections between ocean and land, and their impact on the atmosphere, primarily in the representation of distant climatic connections and lateral fluxes. To achieve this, obstacles to the interoperability of biogeochemical models that represent vegetation, permafrost, rivers, estuaries, and ocean should be identified and resolved to facilitate the flows of carbon, nutrients, and water from one component to the other. Model development within each domain should focus on improving those processes that are susceptible to inflow and control export. For example, better simulation of surface subsidence and hydrological changes following permafrost thaw that affect OC export (Lee et al. 2014). The flows of carbon from land into the ocean should not remain a fixed boundary condition, but considered dynamically in light of the dramatic changes affecting the Arctic following climate change.

## Conclusions

There are many and diverse ways in which the declining arctic cryosphere, as a result of climate change, has put the terrestrial and marine carbon cycles under pressure—as summarized in Table 2. On the one hand, it appears that the uptake of carbon by the Arctic Ocean increased due to sea ice decline—but many processes remain poorly understood and projections are therefore uncertain. The terrestrial environment on the other hand has come under increasing pressure due to higher temperatures and altered precipitation, changes that are likely to be connected to sea ice decline (Bhatt et al. 2014). This can lead to altered plant growth, increased permafrost thaw, and enhanced lateral flows of carbon through freshwater systems and coastal erosion. Large uncertainties remain, however, on the future development of the various components of the arctic carbon cycle, under pressure from permafrost thaw and sea ice decline.

**Table 2:** Terrestrial and oceanic sources and sinks of the Arctic, our knowledge level, and the probable impact of a changing cryosphere on future trends. Updated from Parmentier et al. (2013)

Type of flux	Source/sink	Knowledge level	Expected future trends due to climate change and interactions with the cryosphere
CO_2_ fluxes			
Photosynthesis	Sink	Medium	Shrubification increases uptake, but uncertain future responses to continued increases in summer warmth and changing snow cover—possibly influenced by sea ice decline
Respiration	Source	Medium	Increased emission, but compensated by photosynthesis. Warmer autumns and winters, related to sea ice decline, and permafrost thaw can enhance this source
Net terrestrial flux	Sink	Medium	Dependent on relative responses of photosynthesis and respiration. Permafrost thaw, and tundra fires, can act as a positive feedback
Ocean CO_2_ flux	Sink	Low to medium	Sea ice retreat is likely to enhance the uptake of CO_2_ in coastal shelf regions. Inorganic processes related to sea ice formation/melt are large unknowns, reducing certainty of the future direction of the oceanic CO_2_ sink
Methane fluxes			
Tundra wetlands	Source	Medium	A modest rise in emissions is probably already occurring, with autumn increases most likely connected to sea ice decline
Ocean methane flux	Source	Low	Emission rates still highly uncertain but appear lower than previous estimates. Probably not increasing in the short term due to slow rate of subsea permafrost thaw
Lateral carbon flows			
Dissolved and particulate OC/IC fluxes	Source	Low	Dependent on regional response to thaw, landscape/soil characteristics and relative magnitude of mineralization vs. burial rates
Coastal erosion	Source	Low	Reduced presence of sea ice exposes arctic coast to increased wave action, increasing erosion rates
Ice sheet and glacier OC flux	Source	Low to medium	Increased melt of ice sheets and glacier increases the flow towards the ocean, possibly enhancing the OC flux

To grasp the breadth of consequences following from permafrost thaw, ESMs need to better represent thermokarst processes and the flows of carbon between the land and the ocean. In the ocean, process-focussed research on the fate of the terrestrial carbon is required. Moreover, sea ice decline has occurred faster than CMIP5 models have predicted (Stroeve et al. 2012), and—due to the importance of sea ice extent for the surface energy balance—this suggests that projections of the development of arctic amplification may also be underestimated. Inaccurate representation of marine processes (i.e., sea ice), may therefore affect projections of processes in the terrestrial environment (i.e., ecosystems)—and vice versa (Parmentier et al. 2013; Jeong et al. 2014). This may be particularly of relevance to changes in autumnal emissions, when the warming from sea ice decline is strongest (Parmentier et al. 2015). Since there are many factors connecting the two environments together, a strong effort needs to be made to better understand and improve simulations of linkages between the Arctic Ocean and the land. It has become increasingly clear that the terrestrial and marine environment cannot be considered in isolation to evaluate the future direction of the arctic carbon cycle and associated climate feedbacks.
